# T-cell responses against CD19^+^ pediatric acute lymphoblastic leukemia mediated by bispecific T-cell engager (BiTE) are regulated contrarily by PD-L1 and CD80/CD86 on leukemic blasts

**DOI:** 10.18632/oncotarget.12357

**Published:** 2016-09-30

**Authors:** Judith Feucht, Simone Kayser, David Gorodezki, Mohamad Hamieh, Michaela Döring, Franziska Blaeschke, Patrick Schlegel, Hans Bösmüller, Leticia Quintanilla-Fend, Martin Ebinger, Peter Lang, Rupert Handgretinger, Tobias Feuchtinger

**Affiliations:** ^1^ Department of General Pediatrics, Hematology and Oncology, Children's University Hospital Tübingen, Tübingen, Germany; ^2^ Memorial Sloan Kettering Cancer Center, Center for Cell Engineering, New York, NY, USA; ^3^ Dr. von Hauner Children's Hospital, Ludwig Maximilians University Munich, Munich, Germany; ^4^ Institute of Pathology, University Hospital Tübingen, Tübingen, Germany

**Keywords:** pediatric acute lymphoblastic leukemia, T cells, immune checkpoints, PD-L1, CD80/86, blinatumomab

## Abstract

T-cell immunotherapies are promising options in relapsed/refractory B-precursor acute lymphoblastic leukemia (ALL). We investigated the effect of co-signaling molecules on T-cell attack against leukemia mediated by CD19/CD3-bispecific T-cell engager. Primary CD19^+^ ALL blasts (n≥10) and physiologic CD19^+^CD10^+^ bone marrow precursors were screened for 20 co-signaling molecules. PD-L1, PD-1, LAG-3, CD40, CD86, CD27, CD70 and HVEM revealed different stimulatory and inhibitory profiles of pediatric ALL compared to physiologic cells, with PD-L1 and CD86 as most prominent inhibitory and stimulatory markers. PD-L1 was increased in relapsed ALL patients (n=11) and in ALLs refractory to Blinatumomab (n=5). Exhaustion markers (PD-1, TIM-3) were significantly higher on patients' T cells compared to physiologic controls. T-cell proliferation and effector function was target-cell dependent and correlated to expression of co-signaling molecules. Blockade of inhibitory PD-1-PD-L and CTLA-4-CD80/86 pathways enhanced T-cell function whereas blockade of co-stimulatory CD28-CD80/86 interaction significantly reduced T-cell function. Combination of Blinatumomab and anti-PD-1 antibody was feasible and induced an anti-leukemic in vivo response in a 12-year-old patient with refractory ALL. In conclusion, ALL cells actively regulate T-cell function by expression of co-signaling molecules and modify efficacy of therapeutic T-cell attack against ALL. Inhibitory interactions of leukemia-induced checkpoint molecules can guide future T-cell therapies.

## INTRODUCTION

Acute lymphoblastic leukemia (ALL) is the most common childhood malignancy. Children with standard risk ALL have excellent survival rates with further improvement over the last decades [1]. However, refractory B-precursor ALL and especially relapsed ALL after hematopoietic stem cell transplantation (HSCT) is still associated with a dismal prognosis [2-4]. Immunotherapy and targeted therapy are novel approaches that undergo implementation into treatment strategies in pediatric ALL [3]. The bispecific anti-CD3/CD19 T-cell engager (BiTE) antibody Blinatumomab or T cells expressing chimeric antigen receptors (CARs) can successfully recruit the forces of T cells and guide them against lymphoblastic cells. These polyclonal T cells induce perforin/granzyme-mediated lysis of malignant target cells [5-7] and have the potential to induce hematological remission in adult and pediatric patients with relapsed/refractory B-precursor ALL [2, 8-11]. Despite the encouraging results, it is unknown why T cells could attack malignant blasts in some cases or remained paralyzed in others. There is emerging evidence that loss of co-stimulatory molecules and expression of co-inhibitory molecules have a pivotal role in tumor immune escape [12]. Sustained inhibitory signaling mediated by expression of numerous co-signaling molecules on T cells such as TIM-3, LAG-3, PD-1 or CTLA-4 correlates with a stage of T-cell exhaustion, marked by a reduced T-cell effector function, proliferative potential and cytotoxicity [13, 14]. Since T-cell function is essential for tumor control [15, 16], efforts are made to increase T-cell function and to reverse T-cell exhaustion for induction of a sustained tumor immune surveillance and efficient elimination of malignant cells [17]. Recent advances were achieved by targeting immune escape checkpoints such as CTLA-4 or PD-1 [13, 18, 19]. Antitumor activity of checkpoint blockade was demonstrated in various tumors [18, 20-23], but has not been evaluated in ALL until now. In this study, we examined T-cell attack against pediatric lymphoblastic target cells by analysis of effector-target cell interactions in co-culture experiments with Blinatumomab. As co-inhibitory signaling might interfere with the clinical benefit of T-cell immunotherapy, we examined functional relevance of leukemia-related co-signaling molecules on lymphoblasts for T-cell activity and investigated combined immunotherapy approaches with checkpoint inhibitory antibodies to increase efficacy of T-cell attack against ALL (Table 1). Feasibility of combined treatment with Blinatumomab and PD-1 blocking antibody Pembrolizumab was analyzed in a 12-year-old patient with refractory ALL.

**Table 1:** Summary of co-stimulatory and -inhibitory molecules regulating T-cell responses

Molecule	Synonyme	Cellular expression	Ligand	Main function
PD-1	CD279	T, B, NK, NKT, DC, myeloid cells	PD-L1	inhibition
			PD-L2	inhibition
PD-L1	B7-H1; CD274	T, B, NK, DC, monocytes, macrophages, mast cells	PD1	inhibition
			CD80	inhibition
PD-L2	B7-DC; CD273	T, B, DC, monocytes, macrophages, mast cells	PD1	inhibition
			RGMb	
LAG3	CD223	T, NK	MHC-II	inhibition
			LSECtin	inhibition
HVEM	LIGHTR;	B, T, DC	LIGHT	stimulation
	TNFRSF14;		LT-α	stimulation
	CD270		BTLA	inhibition
			CD160	inhibition
			HSVgD	inhibition
CD160	BY55	T, NK, NKT	HVEM	inhibition
			MHC-I	unclear
BTLA	CD272	T, B, macrophages, DC, NK	HVEM	inhibition
CD200	OX-2	T, B, DC	CD200R	inhibition
Galectin-9		T, DC, granulocytes	TIM3	inhibition
TIM-3	CD366;	T, NK, DC, monocytes, macrophages	Galectin-9	inhibition
	HAVCR2		PtdSer	inhibition
			HMGB1	inhibition
			CEACAM1	inhibition
B7-H3	CD276	T, NK, DC, monocytes, macrophages	?**	inhibition and stimulation*
B7-H4	B7X; B7S1; VTCN1	T, B, DC, monocytes, macrophages	?**	inhibition
CTLA-4	CD152	T, B, DC, monocytes, NK, NKT	CD80	inhibition
			CD86	inhibition
CD86	B7.2	T, B, DC, monocytes, macrophages, mast cells	CD28	stimulation
			CTLA4	inhibition
CD80	B7.1	T, B, DC, monocytes, macrophages, mast cells	CD28	stimulation
			CTLA4	inhibition
			PD-L1	inhibition
CD40	TNFRSF5	B, DC, monocytes, macrophages	CD154	stimulation
CD27	TNFRSF7	T, B, NK	CD70	stimulation
CD70	CD27L	B, T, DC	CD27	stimulation
CD137L	4-1BBL; TNFSF9	DC, monocytes, macrophages, B, T	CD137	stimulation
CD278	ICOS	T, B	ICOS-L	stimulation

* co-stimulatory and co-inhibitory functions have been described, dependent on the context of expression and disease model

** ? Binding of B7-H3 and B7-H4 has not yet been identified

Abbreviations: T: T cells; B: B cells; NK: NK cells; NKT: NKT cells; DC: dendritic cells; PD-1: programmed cell death 1; PD-L: programmed cell death ligand; RGMb: repulsive guidance molecule b; LAG-3: lymphocyte activation gene 3; LSECtin: liver and lymph node sinusoidal endothelial cell C-type lectin; HVEM: herpesvirus entry mediator; TNFRSF: tumor necrosis factor receptor superfamily; LT-α: lymphotoxin-α; BTLA: B- and T-cell lymphocyte attenuator; HSVgD: herpes simplex virus glycoprotein D; TIM-3: T cell immunoglobulin and mucin-3; HAVCR2: Hepatitis A virus cellular receptor 2; PtdSer: phosphatidylserine; HMGB1: high mobility group protein B1; CEACAM1: carcinoembryonic antigen-related cell adhesion molecule 1; VTCN1: V-set domain containing T cell activation inhibitor 1, CTLA-4: cytotoxic T lymphocyte antigen 4; TNFSF: tumor necrosis factor superfamily; ICOS: inducible T-cell co-stimulator

## RESULTS

### T-cell effector function is consistent in leukemia bearing individuals and healthy donors

**Figure 1:**
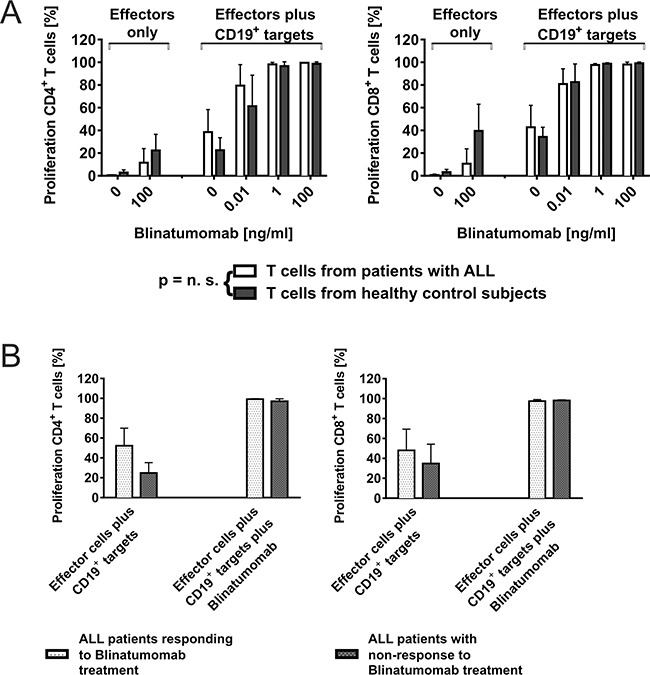
CD4^+^ and CD8^+^ T-cell function can be recruited consistently for attack of CD19^+^ target cells through Blinatumomab **A.** Dose- and target cell-dependent proliferation of T cells from ALL patients and healthy controls after co-incubation with Blinatumomab. PBMC as effectors from patients or healthy controls were incubated with irradiated CD19^+^ target cells (Raji cells; effector/target cell ratio: 10/1) and co-incubated with different concentrations of Blinatumomab. Proliferation of CD4^+^ and CD8^+^ T cells was analyzed by CFSE assay after 5 days. Interexperimental controls were performed with PBMC only, PBMC+Blinatumomab without addition of target cells and PBMC+irradiated Raji without addition of Blinatumomab. PBMC (patients: n=6, controls: n=6); PBMC+Blinatumomab 0.1μg/ml (patients: n=4, controls: n=7), PBMC+Raji (patients: n=6, controls: n=9), PBMC+Raji+Blinatumomab 10pg/ml (patients: n=3, controls: n=8), PBMC+Raji+Blinatumomab 1ng/ml (patients: n=5, controls: n=8), PBMC+Raji+Blinatumomab 0.1μg/ml (patients: n=5, controls: n=8, variable cell numbers due to low cell numbers of patients). **B.** Blinatumomab-induced proliferation of T cells from patients after successful treatment with Blinatumomab (*“in vivo responders*”) is equal to T-cell proliferation of *“non-responders”*. The group of patients depicted in Figure 1A was further grouped in responders (n=3) and non-responders to treatment with Blinatumomab (n=3). Effectors were PBMC from pediatric ALL patients and target cells were irradiated Raji cells. Co-culture experiments were done with addition of Blinatumomab 1ng/ml and 0.1μg/ml.

First, we addressed the question if the difference in T-cell responses against ALL is due to effector- or target-related factors. For analysis of T-cell effector function and proliferation capacity, PBMC from healthy donors were incubated with lymphoblastic cells (Raji) as target cells and exposed to subphysiological, physiological and supraphysiological levels of Blinatumomab. T-cell function was analyzed in terms of proliferation, CD107a expression, cytokine production and GrB/Perforin expression - with variable E/T cell ratios and incubation times (Figures 1 and Supplementary Figure S1). In all cases, T-cell function was shown to be strictly target cell- and dose-dependent. After stimulation with physiological *in vivo* serum levels of 100pg/ml-1ng/ml Blinatumomab [24], high T-cell proliferation rates were induced, as determined by flow cytometry after 5 days – with a mean CD4^+^ T-cell proliferation of 97.1%±3.5 (mean±SD, n=10) after stimulation with Blinatumomab 1ng/ml (Supplementary Figure S1A). In contrast, proliferation of T cells was low when PBMC were incubated with high dose of 0.1μg/ml Blinatumomab without addition of target cells or with Raji cells without addition of Blinatumomab (Figures 1 and Supplementary Figure S1A). Despite variable E/T cell ratios, different incubation times and doses of Blinatumomab, there was no significant difference in analyzed T-cell function between different donors (Figures 1 and Supplementary Figure S1). Analysis of different cell populations confirmed dose-dependent recruitment of T cells as effector cells whereas NK-cell activity remained independent of Blinatumomab (Supplementary Figure S1A).

To analyze whether effector cells or target cells determine T-cell attack against leukemic blasts, we compared T-cell function from patients responding to Blinatumomab treatment, non-responders and healthy donors. Results of T-cell proliferation and CD107a expression were compared within the group of patients (*in vivo* responders vs *in vivo* non-responders) and to T-cell function of healthy donors (Figures 1B and Supplementary Figure S1D). Patients and controls both showed target cell- and dose-dependent CD107a expression and proliferation of T cells as detected by CFSE assay and flow cytometry. There was neither a significant difference of T-cell function between responders (n=3) and non-responders (n=3), nor between patients and healthy donors (Figure 1), with a mean CD4^+^ T-cell proliferation of 98.2%±1.7 (mean±SD, n=5) among patients as compared to 96.7%±3.8 (mean±SD, n=8) among controls under 1ng/ml Blinatumomab. As *in vivo* responders and non-responders to treatment with Blinatumomab both showed similar results regarding induced T-cell function (Figure 1B), there was no correlation of *in vivo* and *in vitro* results when irradiated Raji cells were used as target cells.

### Leukemia-related co-inhibition and co-stimulation is crucial for T-cell function against lymphoblasts

**Figure 2:**
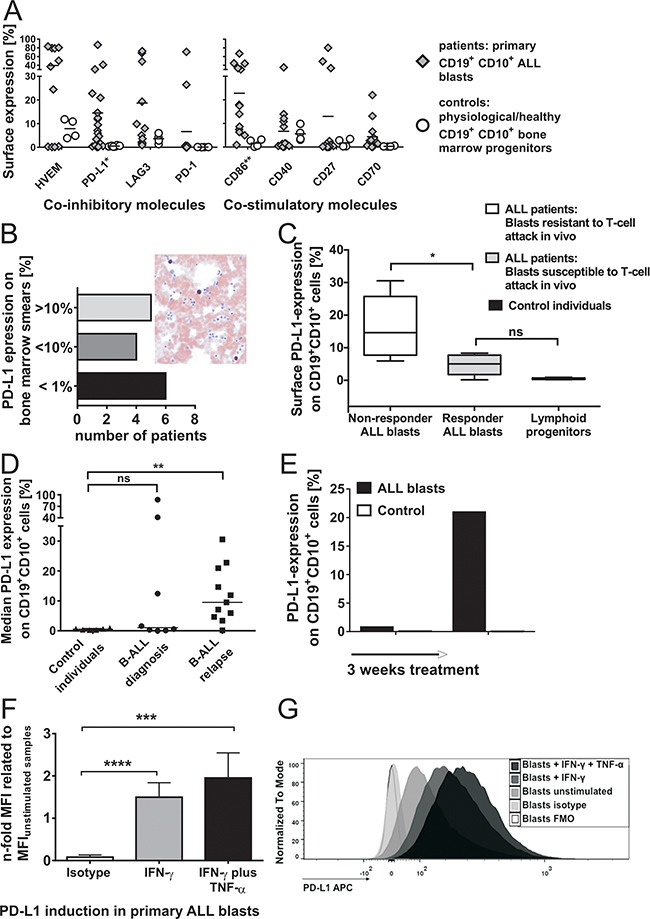
**A.** Surface expression of co-inhibitory and co-stimulatory molecules on CD19^+^CD10^+^ cells in the bone marrow of patients and control individuals (without malignancies). Surface expression of inhibitory molecules (left plot) PD-L1, LAG-3 and PD-1, of the bifunctional molecule HVEM and of co-stimulatory molecules (right plot) CD86, CD40, CD27 and CD70 on CD19^+^CD10^+^ bone marrow cells of patients (n≥11) as compared to controls (n≥4) was determined *ex vivo* by flow cytometry. Results with primary blasts prove an interindividual distinguishable pattern of inhibitory and stimulatory markers on leukemia cells. Each symbol represents an individual sample and the mean is indicated. Differences are statistically significant for PD-L1 (*p < 0.05) and CD86 (**p < 0.01, Mann-Whitney test). **B.** Immunohistochemical staining of PD-L1 expression on patients' bone marrow blasts. PD-L1 expression was analyzed on patients' bone marrow blasts by immunohistochemistry. The plot demonstrates the number of patients with their corresponding frequencies of immunohistochemical PD-L1 expression (grouped in patients with PD-L1 expression >10%, 1-10% and <1%). The right figure shows a representative example of immunohistochemical PD-L1 staining on one patient's ALL bone marrow blasts which was additionally confirmed by flow cytometry. **C.** Surface expression of PD-L1 on CD19^+^CD10^+^ cells from responders as compared to non-responders to Blinatumomab-treatment. PD-L1 expression on CD19^+^CD10^+^ cells of *in vivo* responders (n=4) and non-responders (n=5) to treatment with Blinatumomab and on CD19^+^CD10^+^ bone marrow cells of controls (n=6) was determined by extracellular antibody staining and flow cytometry. Box and whiskers show min/max and median (*p < 0.05, Mann-Whitney test). **D.** Surface expression of PD-L1 on CD19^+^CD10^+^ cells at primary diagnosis as compared to relapse. Median PD-L1 expression on ALL blasts of patients at primary diagnosis (n=8) compared to median PD-L1 expression on ALL blasts of relapsed/refractory patients (n=11) and to median PD-L1 expression on physiologic CD19^+^CD10^+^ bone marrow cells of controls (n=6), as determined by flow cytometry. Each symbol represents an individual sample and the median is indicated. ** p < 0.01 between relapse and controls and p = ns between controls and initial diagnosis using Mann-Whitney test. **E.** Induction of PD-L1 in leukemia cells *in vivo* through treatment. The plot demonstrates initial absence of PD-L1 surface expression on ALL blasts of one patient and development of PD-L1^+^ ALL blasts refractory to therapy in the further treatment course (control = isotype control). **F. & G.** Induction of PD-L1 in primary pediatric ALL blasts through inflammatory TH1 cytokines. Induced PD-L1 surface expression was determined in different ALL patients (n=7) after 42-44h incubation with IFN-γ or IFN-γ and TNF-α as compared to unstimulated samples and FMO (Fluorescence Minus One)/ isotype control. Mean Fluorescence Intensity (MFI) of PD-L1 on CD19^+^CD10^+^ blast cells after stimulation with IFN-γ, after stimulation with IFN-γ and TNF-α and of isotype staining were compared to MFI of unstimulated patients' samples. Bars show mean and SD of 7 independent experiments. ***p < 0.001, ****p < 0.0001, paired t test. In Figure G, an example is shown of TH1-induced PD-L1 expression on one patient's bone marrow blasts after incubation with IFN-γ or IFN-γ and TNF-α as compared to unstimulated patient's blasts, isotype and FMO control.

For analysis of bone marrow blasts, at least 10 pediatric ALL patients were screened for expression of a variety of co-inhibitory and co-stimulatory molecules by flow cytometry (Table 1). Results were compared to expression pattern on physiologic CD19^+^CD10^+^ cells in healthy bone marrow samples (Figures 2A and Supplementary Figure S2A). We especially aimed to identify markers with interindividual differences as these molecules might be candidates explaining functional differences. *Ex vivo* expression pattern of inhibitory molecules PD-L1, LAG-3 and PD-1, the bi-functional molecule HVEM and of co-stimulatory molecules CD86, CD40, CD27 and CD70 revealed interindividual differences on patients blasts' as compared to consistent low or absent expression on CD19^+^CD10^+^ cells of controls (Figure 2A). The most prominent inhibitory marker on primary pediatric blasts was PD-L1. The stimulatory marker CD86 was significantly higher expressed on malignant lymphoblastic cells compared to physiologic CD19^+^CD10^+^ bone marrow precursors. Expression pattern of co-signaling molecules BTLA, CD80, PD-L2, B7H3, B7H4, CD160, Galectin9, CD137L, CD278, CTLA-4 and TIM-3 was similar for patients and controls, with uniform low or absent expression on the surface of CD19^+^CD10^+^ bone marrow cells. The co-inhibitory molecule CD200 was expressed in high levels on patients' blasts (mean±SD CD200 expression= 90%±17) and on controls, with no significant intra- and interindividual difference between the two groups (Supplementary Figure S2A).

PD-L1 expression was significantly higher on patients' blasts of non-responders to Blinatumomab (median PD-L1 expression: 14.6%, n=5) as compared to responders (median PD-L1 expression: 5.0% n=5) and to controls (p = 0.0022; n=6) (Figure 2C). Median PD-L1 surface expression was higher on patients' ALL blasts at relapse (median PD-L1 expression: 9.5%; n=11) as compared to patients with primary diagnosis (median PD-L1 expression: 1.1%; n=8) and to controls (median PD-L1 expression: 0.49%, n=6) (Figure 2D). Samples for analysis of PD-L1 surface expression at diagnosis and relapse did not originate from the same patients. The findings of increased PD-L1 surface expression at relapse compared to expression levels at diagnosis might contain an intrinsic element of heterogeneity and therefore have to be confirmed in further analyses of leukemic blasts originating from the same patients.

Expression of PD-L1 on ALL blasts could be upregulated during the course of treatment and was shown to be inducible after 24-44h stimulation with Th1 cytokines TNF-α and IFN-γ (Figures 2E, 2F and 2G). Expression pattern of co-signaling molecules on CD19^+^CD10^+^ cell lines demonstrated preponderance of co-stimulatory molecules on target cells susceptible to T-cell attack (Raji, Supplementary Figure S3) as compared to less immunogenic lymphoblasts (NALM-6, NALM-16 and MHH-CALL-4) with predominantly absent co-stimulatory molecules (data not shown). PD-L1 expression on B lymphoblastic cells (e.g. NALM-16) was shown to be inducible after stimulation with Th1 cytokines IFN-γ or TNF-α plus IFN-γ. Thus, surface expression of co-signaling molecules on CD19^+^CD10^+^ cells can strongly differ between individuals. A range of co-signaling molecules are variably expressed on patients' blasts with gradual differences in expression levels and concomitant absence on CD19^+^CD10^+^ bone marrow cells of controls. In contrast, analysis of intracellular PD-L1 expression showed consistently high expression in B-cell malignancies (cell lines) and patients' ALL blasts. Immunohistochemical analysis of PD-L1 expression on bone marrow smears of patients' blasts confirmed interindividual differences in expression levels of PD-L1 (Figure 2B).

### Expression level of T-cell exhaustion markers is higher among leukemia patients as compared to healthy controls and is upregulated during T-cell attack against leukemia

**Figure 3:**
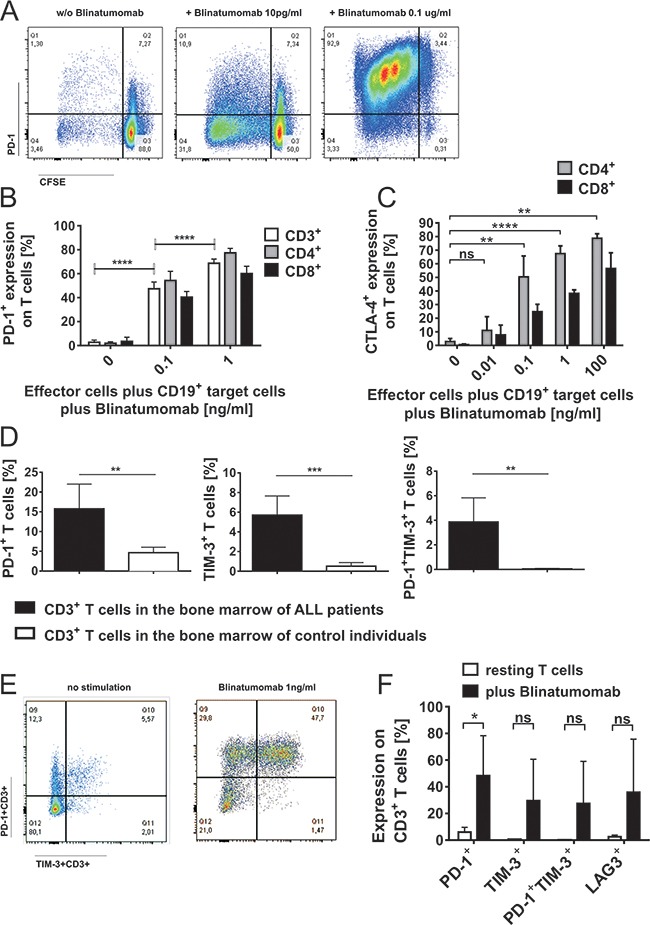
Induction of PD-1 and CTLA-4 on T cells during attack of malignant lymphoblast cells mediated by Blinatumomab **A.** PBMC of healthy donors were incubated with irradiated Raji cells (effector/target cell ratio: 10/1) and stimulated with different concentrations of Blinatumomab for 48 hours. Flow-cytometric analysis of proliferation (determined by CFSE) and PD-1 expression on CD3^+^ T cells incubated with irradiated Raji cells without addition of Blinatumomab (left plot) and after 48h-incubation with 10pg/ml (central plot) and 0.1μg/ml Blinatumomab (right plot). **B.** Dose-dependent PD-1 expression on CD3^+^, CD4^+^ and CD8^+^ T cells of healthy donors (n=7 after stimulation with Blinatumomab, n=6 without Blinatumomab-stimulation) after 48h-incubation of PBMC with irradiated Raji cells and addition of 100pg/ml or 1ng/ml Blinatumomab. Bars show mean and SD. ****p<0.0001, paired t test of results from CD3^+^ cells. **C.** Dose-dependent expression of CTLA-4 (intracellular) after 48-72h incubation of PBMC (healthy donors) with irradiated Raji cells (E/T=10/1) and stimulation with Blinatumomab. PBMC+irradiated Raji cells: n=5, PBMC+irradiated Raji cells+Blinatumomab 10pg/ml: n=4, PBMC+irradiated Raji cells+Blinatumomab 100pg/ml: n=4, PBMC+irradiated Raji cells+Blinatumomab 1ng/ml: n=5, PBMC+irradiated Raji cells+Blinatumomab 0.1μg/ml: n=3. Bars show mean and SD. **p < 0.01, ****p < 0.0001, paired t test of results from CD4^+^ cells. **D. & E.** Surface expression of PD-1, TIM-3 and PD-1^+^TIM-3^+^ on CD3^+^ T cells in the bone marrow of ALL patients. Single and double positive expression of exhaustion markers PD-1 and TIM-3 on CD3^+^ T cells in bone marrow samples of patients (n=6) as compared to controls (n=4) **D.** Representative flow cytometric analysis of PD-1 and TIM-3 expression on CD3^+^ T cells of one patient without stimulation and after 48h stimulation with 1ng/ml Blinatumomab **E.** Bars represent data from independent experiments and mean and SD are indicated. **p < 0.01, ***p < 0.001, unpaired t test. **F.** Upregulation of PD-1, TIM-3 and LAG-3 on T cells during attack of malignant lymphoblast cells mediated by Blinatumomab. Expression of PD-1, TIM-3, LAG-3 and PD-1^+^TIM-3^+^ (mean±SD) on patients' bone marrow infiltrating CD3^+^ T cells without Blinatumomab and after 48h-incubation with 1ng/ml Blinatumomab (n=5). Bars show mean and SD of 5 independent experiments. *p < 0.05, paired t test.

Since target cell analyses demonstrated differences in the expression of co-signaling molecules, we analyzed effector T cells for expression of the corresponding binding partners. Blinatumomab-induced recognition of target cells led to a dose- and target cell-dependent increase in expression of T-cell exhaustion markers CTLA-4, PD-1, TIM-3 and LAG3 (Figures 3A-3C, 3E-3F and Supplementary Figure S2B). Mean PD-1 expression of CD4^+^ T cells was 1.9%±1.3 (mean±SD, n=6) after 48h-incubation of PBMC with irradiated Raji cells and could be significantly increased up to 77.4%±3.8 (mean±SD, n=7) after addition of Blinatumomab 1ng/ml (Figure 3B). Dose-dependent induction of PD-1 expression could be confirmed at different effector/target cell ratios (data not shown). Surface expression of exhaustion markers PD-1^+^, TIM-3^+^ and PD-1^+^TIM-3^+^ on CD3^+^ T cells in the bone marrow was significantly higher among leukemia patients as compared to physiological bone marrow T cells (Figure 3D). Expression of exhaustion markers PD-1, LAG-3 and TIM-3 on T cells could be further upregulated in leukemia patients after addition of Blinatumomab (Figures 3E and 3F).

### Leukemia cells (target cells) control Blinatumomab-induced T-cell effector function

**Figure 4:**
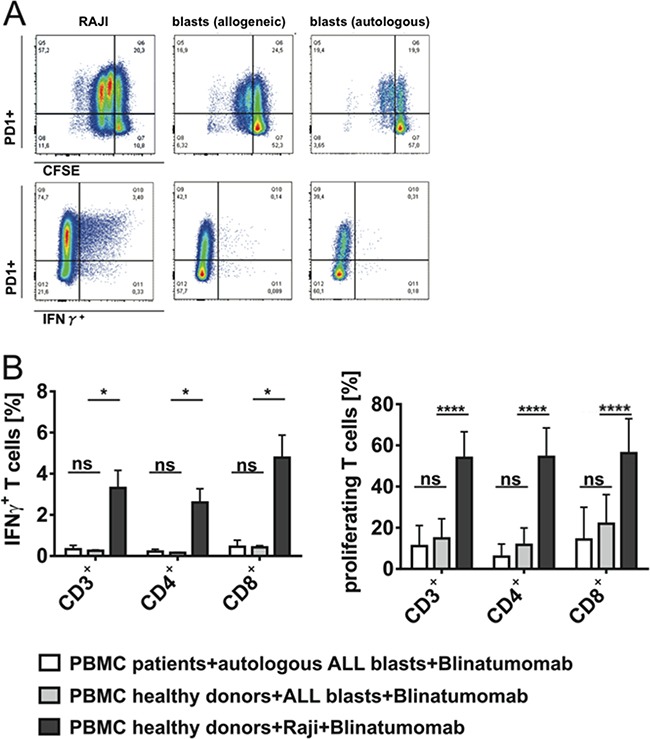
Target cell-dependent proliferation and IFN-γ secretion of T cells under stimulation with Blinatumomab PBMC of patients or healthy donors were incubated with irradiated Raji cells or patients' blasts and 1ng/ml Blinatumomab for 48h. **A.** Flow cytometric analysis of target cell-dependent proliferation and effector capacity of CD3^+^ T cells. Blinatumomab-induced PD-1 expression, proliferation and IFN-γ secretion of CD3^+^ T cells are demonstrated after incubation of one donor's PBMC with irradiated Raji cells (left plots) or patient's blasts (central plots) as target cells and after incubation of the patient's PBMC with autologous blasts (right plots) and stimulation with Blinatumomab 1ng/ml. **B.** IFN-γ secretion (n=3) and proliferation (n=7) of CD3^+^, CD4^+^ and CD8^+^ T cells of healthy donors as compared to patients after 48h incubation of PBMC with irradiated Raji cells or patients' blasts as target cells and stimulation with 1ng/ml Blinatumomab. Bars indicate mean and SD. *p < 0.05, ****p < 0.0001, paired t test.

Next, we analyzed how different target cells can modify functional T-cell characteristics during T-cell attack (numbers are given as mean±SD). T-cell proliferation capacity and IFN-γ secretion were markedly reduced when PBMC of healthy donors were incubated with primary patients' blasts instead of Raji cells under the same experimental conditions (Figure 4). Incubation of PBMC from healthy donors with irradiated Raji cells led to a mean IFN-γ secretion of 3.3%±0.9 (n=3) and to a mean T-cell proliferation of 50.5%±12.0 (n=7) among CD3^+^ T cells under 48h-presence of Blinatumomab. In contrast, incubation of the same donor PBMC with primary patients' ALL blasts led to a significant reduction in IFN-γ secretion and T-cell proliferation (0.3%±0.0 IFN-γ^+^CD3^+^ T cells (n=3) and CD3^+^ T-cell proliferation of 12.7%±8.6; n=7). Similar results were obtained when patients' PBMC were incubated with their autologous ALL blasts – with a mean IFN-γ secretion of 0.3%±0.2 (n=3) and a mean T-cell proliferation of 9.86%±8.67 (n=7) among CD3^+^ T cells. Besides, target cell-dependent differences in T-cell function could be demonstrated when PBMC of the same donors were incubated with different cell lines as target cells (Supplementary Figure S2B). We could confirm that target-cell related co-signaling factors determine the effect of T-cell attack against leukemic blasts by luciferase-based cytotoxicity assays. In order to evaluate the impact of co-signaling molecules on Blinatumomab-mediated cytolytic activity, we consistently used GFP/luciferase expressing NALM-6 as target cells as well as their retrovirally transduced sublines expressing either PD-L1 or CD80 (cells were kindly provided by Dr. Michel Sadelain, MSKCC, New York) (Figure 6A). We could show that cytolytic activity mediated by Blinatumomab was significantly decreased under inhibitory influence of PD-L1 and significantly increased when target cells provided co-stimulatory signaling via the CD80-CD28 axis (Figure 6).

**Figure 5:**
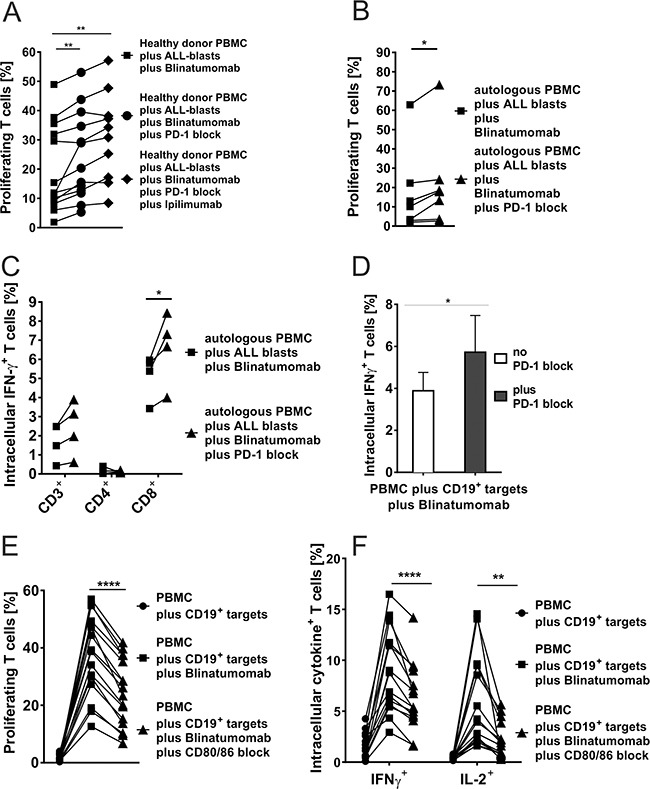
A-D. Blinatumomab-induced IFN-γ secretion and proliferation of T cells after blocking the PD-1–PD-L or CD80/CD86–CD28 axis **A.** Flow cytometric analysis of proliferation of T cells from healthy donors after 48h-incubation with irradiated bone marrow leukemia blasts (E:T ratio 2:1) and 1ng/ml Blinatumomab as compared to additional use of PD-1 blocking antibody (n=13) or addition of PD-1 blocking antibody and CTLA-4 blocking antibody Ipilimumab (n=10). CD3^+^ T-cell proliferation was significantly increased through blockade of both checkpoint molecules (PD-1 and/or CTLA-4). Both, CD4^+^ and CD8^+^ T cells showed equivalent responses (data not shown). Each symbol represents one sample and results from one individual are linked by a line (**p < 0.01, paired t test). **B.** Proliferation (n=7) of patients' CD3^+^ T cells (CD4^+^ and CD8^+^ data not shown) after 48h-incubation of autologous PBMC with autologous bone marrow blasts (E:T ratio 2:1) and 1ng/ml Blinatumomab as compared to additional use of PD-1 blocking antibody. Blockade of PD-1 significantly increased T-cell proliferation (*p < 0.05, paired t test). **C.** IFN-γ secretion of CD3^+^, CD4^+^ and CD8^+^ T cells (n=4) after 48h-incubation of autologous PBMC with autologous bone marrow blasts (E:T ratio 2:1) and 1ng/ml Blinatumomab as compared to additional use of PD-1 blocking antibody which further increased T-cell activation (*p < 0.05, paired t test). **D.** IFN-γ secretion of CD3^+^ T cells (n=6) after 48h-incubation of autologous PBMC with irradiated Raji cells (E:T ratio 10:1) and Blinatumomab (100pg/ml) with and without addition of PD-1 blocking antibody. Gating on CD4^+^ and CD8^+^ T cells showed identical results as well as different E:T ratios (100:1) and Blinatumomab concentrations (1ng/ml) (data not shown; *p < 0.05, paired t test). **E. & F.** Blockade of the co-stimulatory markers CD80 and CD86 significantly reduced Blinatumomab-induced effector function and proliferation capacity of T cells. PBMC of healthy donors were incubated with CD80^+^CD86^+^ irradiated Raji cells (E:T ratio 2:1) for 48h and stimulated with 1ng/ml Blinatumomab. IL-2 secretion, IFN-γ secretion (n=15) and proliferation (n=16) were analyzed without antibody blockade and after addition of blocking antibodies against CD80 and CD86. CD4^+^ and CD8^+^ T-cells showed equivalent results (data not shown; **p < 0.01, ***p < 0.001, ****p < 0.0001, paired t test).

**Figure 6:**
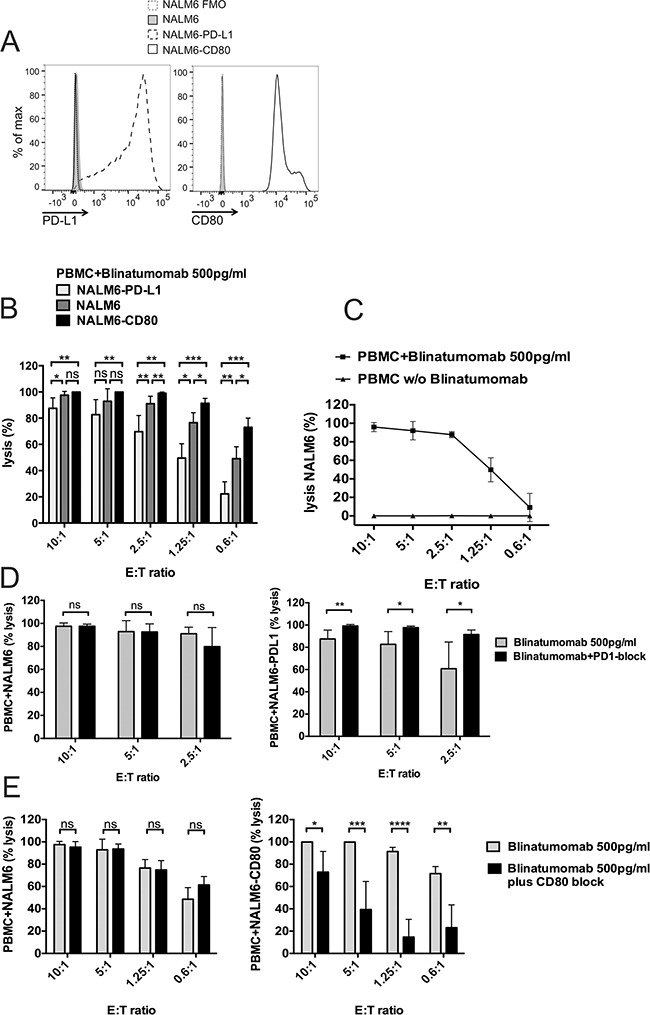
Target-cell dependent cytotoxicity mediated by Blinatumomab PBMC were incubated with Blinatumomab and target cells at different effector:target (E:T) ratio. NALM-6 expressing firefly luciferase-GFP (NALM-6), NALM-6 expressing firefly luciferase-GFP and PD-L1 (NALM-6-PD-L1) or NALM-6 expressing firefly luciferase-GFP and CD80 (NALM-6-CD80) served as target cells. **A.** Representative FACS plots demonstrating expression of CD80 and PD-L1 on respective target cells. **B.** PBMC were stimulated with 500pg/ml Blinatumomab and cytolytic capacity against the targets NALM-6, NALM-6-CD80 and NALM-6-PDL1 were compared after 46 hours of co-culture (n≥4) by bioluminescence assay. Lysis was compared to lysis of the same conditions without addition of Blinatumomab. Results are pooled data from three independent experiments performed in duplicate or triplicate wells. Data are means ± SD. *p < 0.05, **p < 0.01, ***p < 0.001, paired t test. **C.** Cytotoxic activity of PBMC against NALM-6 after stimulation with Blinatumomab 500pg/ml or without Blinatumomab (n≥5). Results are pooled data from three independent experiments performed in duplicate or triplicate wells. Data are means ± SD. **D.** Effect of PD1-blockade and **E.** CD80-blockade on Blinatumomab-mediated cytotoxicity. PBMC were incubated with indicated target cells (NALM-6, NALM-6-PDL1 or NALM-6-CD80) and 500pg/ml Blinatumomab in the absence or presence of blocking antibodies against PD-1 (D) or CD80 (E) at different effector:target (E:T) ratios for 46h. Results are pooled data from three independent experiments performed in duplicate or triplicate wells. Data are means ± SD (n≥6 for PD1 blockade and n≥4 for CD80 blockade). *p < 0.05, **p < 0.01, ***p < 0.001, ****p < 0.0001, paired t test. Lysis is compared to lysis of the same conditions without Blinatumomab.

### T-cell responses against leukemia are inhibited by blockade of CD86/CD80-signaling and increased by blockade of PD-L1 and CTLA-4 signaling pathways

In order to analyze influence of co-signaling interactions on T-cell function, we performed functional T-cell assays with addition of blocking antibodies against either CD80 and CD86 to block co-stimulatory CD80/CD86-CD28 interactions or against PD-1 and/or CTLA-4 for blockade of inhibitory PD-1–PD-L or CTLA-4–CD80/CD86 signaling (numbers are given as mean±SD; Figure 5). For analysis of CD80/CD86-CD28 blockade (Figures 5E and 5F), PBMC of healthy donors (n≥15) were incubated with irradiated CD80^+^CD86^+^ Raji cells and stimulated with Blinatumomab 1ng/ml for 48 hours. Addition of CD80/CD86-blocking antibodies led to a significant decrease in T-cell proliferation, IL-2 and IFN-γ secretion when Raji cells were used as target cells whereas control experiments with CD80^−^CD86^−^ NALM-6 cells or addition of anti-CD80/CD86 antibodies to unstimulated co-cultures of PBMC and Raji cells showed no significant differences. Co-culture experiments with PBMC and Raji cells showed a reduction of CD3^+^ T-cell proliferation from 37.8%±14 under stimulation with 1ng/ml Blinatumomab to 24.6%±11.6 after addition of CD80/CD86 blocking antibodies (n=16, Figure 5E). In order to examine the influence of co-inhibitory signals mediated by the PD-1–PD-L and CTLA-4-CD80/CD86 axis, PBMC of healthy donors or patients were incubated with irradiated Raji cells or patients' blasts and stimulated with Blinatumomab in the presence or absence of PD-1 and/or CTLA-4 blocking antibody. Experiments with Raji cells as target cells demonstrated significant increase of IFN-γ secretion by CD4^+^ and CD8^+^ T cells under PD-1 blockade at different E/T ratios and concentrations of Blinatumomab (Figure 5D). When patients' ALL blasts were used as target cells, proliferation of T cells could be significantly increased under PD-1 blockade or under combined blockade of PD-1 and CTLA-4 (Figure 5A): CD3^+^ T-cell proliferation was 19.8%±14.9 after stimulation of PBMC with irradiated ALL blast cells and 1ng/ml Blinatumomab (n=13) and was increased to a mean CD3^+^ T-cell proliferation of 24.4%±15 after addition of PD-1 blocking antibody and of 31.1%±15.1 after PD-1 and CTLA-4 antibody blockade. These results could be confirmed for patients' PBMC and for PBMC of healthy donors as effector cells (Figures 5A-5B). IFN-γ secretion was also increased by blocking PD-1–PD-L interactions and reached statistical significance for CD8^+^ T cells (Figure 5C). Moreover, Blinatumomab-mediated cytotoxicity could be significantly increased under PD-1 blockade when PD-L1-expressing NALM-6 luciferase-GFP cells served as targets. Consistently, cytolytic capacity was significantly decreased by inhibition of co-stimulatory CD28-CD80 interactions when CD80-transduced NALM-6 luciferase-GFP cells were used as targets. There was no effect of PD-1 or CD80 blockade when PD-L1^−^CD80^−^ NALM6 luciferase-GFP cells served as targets (Figures 6D and 6E and there was no relevant allo-reactive cytotoxicity in control experiments without addition of Blinatumomab (Figure 6C). Therefore, we proved that stimulatory and inhibitory interactions between effector cells and lymphoblastic target cells guide T-cell function against leukemia through CD80/86–CD28/CTLA-4 as well as PD-1–PD-L interactions.

### Safety and feasibility of combined treatment with Blinatumomab and PD-1 blocking antibody Pembrolizumab

**Figure 7:**
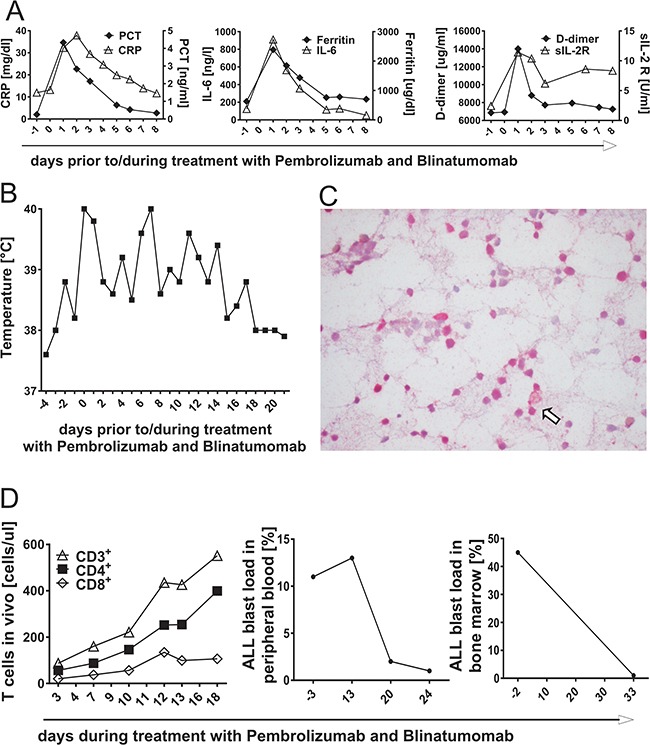
Inflammatory response during T-cell attack of acute lymphoblastic leukemia *in vivo*, mediated by treatment with PD-1 blocking antibody Pembrolizumab and Blinatumomab **A. & B.** A 12-year-old girl suffering from refractory ALL was treated with combined therapy of Pembrolizumab and Blinatumomab. Blood results of inflammatory parameters C-reactive protein (CRP), Procalcitonin (PCT), ferritin, D-dimer, soluble IL-2 receptor (sIL-2R) and interleukin-6 (IL-6) as well as fever chart prior to and during combinatory treatment with Blinatumomab and Pembrolizumab. Day 0 = day of treatment start with Blinatumomab, day -1 = first application of Pembrolizumab. **C.** Immunohistochemical PD-L1 staining of the patient's bone marrow prior to treatment start. Immunohistochemistry revealed high percentage of PD-L1^+^ ALL blasts. The arrow shows one example of PD-L1^+^ bone marrow blasts. **D.** T-cell expansion in peripheral blood and blast load after treatment start with Pembrolizumab and Blinatumomab. The left plot demonstrates proliferation of CD3^+^, CD4^+^ and CD8^+^ T cells after application of Pembrolizumab and during treatment with Blinatumomab as determined by flow cytometry. The middle plot shows the course of ALL blast load in peripheral blood and the right plot the course of ALL blast load in bone marrow under combined treatment with Pembrolizumab and Blinatumomab.

Combined treatment approach with Blinatumomab and PD-1 blocking antibody Pembrolizumab was applied to a 12-year-old girl with refractory ALL after 2^nd^ HSCT and previous non-response to monotherapy with Blinatumomab. Immunohistochemical staining prior to treatment start revealed PD-L1 expression of nearly all bone marrow blasts (Figure 7C). Pembrolizumab was administered at a dose of 1mg/kg body weight one day after transfusion of CD45RA-depleted T cells from her haploidentical stem cell donor. Blinatumomab was added one day later at a dose of 15μg/m^2^/d without previous steroid application. Further applications of Pembrolizumab were given after 18 and 29 days. Additional CD45RA-depleted T cells from her HSCT donor were infused at time of third Pembrolizumab administration. Leukemia load prior to treatment start revealed 45% bone marrow blasts. Treatment with Blinatumomab and Pembrolizumab was safe without acute toxicities, but with expected inflammatory response associated with high fever and transient increase of inflammatory parameters IL-6, soluble IL-2 receptor, CRP, procalcitonin, D-dimer and ferritin (Figures 7A and 7B). Antileukemic response was confirmed by flow cytometric detection of T-cell expansion and reduction of blast load in peripheral blood under treatment (Figure 7D). *Ex vivo* analysis predicted a low effector T-cell function against patient's ALL blasts (co-culture experiment: T cells plus primary blasts plus Blinatumomab), but an increased T-cell function after addition of PD-1 blocking antibody (data not shown). Bone marrow analysis 34 days after treatment start showed morphological remission of ALL blasts to <5% (Figure 7D, right plot). Treatment with Blinatumomab ended 2 days later and there was no further administration of Pembrolizumab. The patient is still alive, although relapse occurred 2 months after this combined treatment.

## DISCUSSION

Despite considerable improvement in survival rates of pediatric patients with acute B-lymphoblastic leukemia (B-ALL), relapsed and refractory ALL is still associated with a poor prognosis [3, 4]. Novel approaches focus on T-cell therapy, e.g. *ex vivo* genetic modification of T cells to express chimeric antigen receptors (CARs) targeting leukemic antigens or by antibody-mediated activation of patients' T cells [25]. The CD19/CD3-bispecific T-cell engager antibody Blinatumomab is a successful example of antibody-based T-cell treatment with the potential to induce long-term clinical benefits in patients with refractory B-ALL [8, 10, 11]. Despite this promising data, some patients do not respond to these novel immunotherapeutic approaches. It is therefore important to uncover parameters that determine the fate of a T-cell response against leukemia cells. With functional assays we addressed the question whether factors on the target side (ALL blasts) or effector side (T cells) are responsible for differences in the Blinatumomab-mediated interaction between leukemia and T cells. Significant differences in T-cell function were found to be target-cell dependent whereas *ex vivo* T-cell function itself was consistent between patients with clinical response to Blinatumomab treatment and non-response. To uncover the mechanisms how pediatric ALL blasts manipulate T-cell responses, we investigated phenotypic and functional relevance of stimulatory and inhibitory co-signaling in primary blasts. Use of different target cells characterized by variable expression of co-signaling molecules on their cell surface resulted in differences of T-cell function. Co-signaling molecules PD-L1, PD-1, LAG-3, CD40, CD86, CD27, CD70 and HVEM showed significantly different expression on primary pediatric ALL blasts (n≥10) as compared to physiologic CD19^+^CD10^+^ bone marrow cells, confirming inter-individual differences in immune escape of ALL blasts. We demonstrate that co-signaling molecules influence T-cell attack against leukemic blasts and have a pivotal role for effector-target cell interactions in pediatric ALL. Most prominent T-cell inhibitory and stimulatory markers expressed on pediatric ALL blasts were PD-L1 and CD86 respectively. T-cell proliferation, cytokine secretion and cytotoxicity were increased when target cells showed high expression of mainly co-stimulatory molecules (e.g. CD80^+^CD86^+^) as compared to target cells with absence of co-stimulatory molecules or predominant expression of co-inhibitory molecules. Blockade of inhibitory PD-1–PD-L1 interactions and combined blockade of PD-1–PD-L1 and CTLA-4-CD80/86 interactions could further enhance effector T-cell function whereas blockade of the CD28-CD80/CD86 pathway led to significant reduction of T-cell response against ALL.

Flow cytometric and immunohistochemical staining confirmed variable PD-L1 expression patterns on patients' bone marrow blasts. All patients with clinical non-response to Blinatumomab-treatment showed high surface expression of PD-L1 on their leukemic blasts. Furthermore, median PD-L1 expression was higher on ALL blasts of patients at relapse as compared to patients at primary diagnosis. A role of PD-L1 in resistance to Blinatumomab was suggested for the first time in a recent case report of a patient with refractory B-precursor ALL [26]. Our data demonstrate functional relevance of leukemic PD-L1 expression for T-cell inhibition and provide further insight into relevant mechanisms of co-signaling pathways for effector-target cell interactions, contributing to immune escape of pediatric ALL. As shown in other malignancies and models [12, 27-29], we now demonstrate that PD-L1 surface expression can also be dynamic in ALL. PD-L1 expression could be induced by stimulation with Th1 cytokines IFN-γ and TNF-α and could be upregulated in the course of disease.

Expression of exhaustion markers PD-1 and TIM-3 was significantly increased on patients' bone marrow infiltrating T cells as compared to healthy donors. Co-expression of both molecules has been shown to represent tumor infiltrating lymphocytes with the most exhausted T-cell phenotype as defined by dysfunctional proliferation potential and cytokine secretion which could be restored by combined antibody blockade [14, 30]. Expression of inhibitory checkpoint molecules PD-1, CTLA-4, LAG-3 and TIM-3 [14] was induced and upregulated in a dose-dependent fashion through Blinatumomab. Concentrations of Blinatumomab were correlated to those achievable *in vivo* under continuous infusion [31, 32]. Our findings thus indicate that T-cell attack against ALL by itself induces suppressive interference of inhibitory regulators on leukemic ALL blasts with their binding partners expressed on T cells.

Based on this data, a 12-year-old patient with a pre-B-ALL refractory to Blinatumomab received combined treatment with Blinatumomab and PD-1 blocking antibody Pembrolizumab. The therapy induced an inflammatory response, was feasible and safe without acute toxicities. Flow cytometric analysis confirmed antileukemic response with detectable T-cell expansion and reduction of blast load in peripheral blood. Bone marrow analysis at the end of the therapy with Pembrolizumab and Blinatumomab (after a treatment period of 34 days) showed reduction of leukemic blasts from 45% to 1%. The case report shows that the combination of lymphocyte infusion, Blinatumomab and Pembrolizumab led to a successful induction of *in vivo* T-cell expansion and to reduction of ALL blasts. However, it does not allow a direct mechanistic conclusion to determine the relative contribution of the three components. Two of the three components have been shown unsuccessful before (donor cell infusion and Blinatumomab).

In conclusion, we examined the role of immune regulators as potential immunotherapeutic targets in pediatric ALL. We show that leukemic blasts can differentially express co-signaling molecules on their surface and that regulation of T-cell activation/inhibition by co-signaling molecules impacts effector-target cell interactions in pediatric ALL. Blinatumomab-induced inhibitory interactions between T cells and their counterparts expressed on target cells - such as PD-1-PD-L signaling or loss of co-stimulation through CD80 or CD86 - might contribute to *in vivo* resistance to therapy. Combined treatment approaches with checkpoint blocking antibodies could thus be a promising therapeutic strategy in pediatric refractory/relapsed B-ALL in order to increase antitumor T-cell activity. The efficacy of this treatment approach will have to be evaluated in larger patient cohorts in future studies. Immunotherapy targeting further negative regulators (e.g. CTLA-4, LAG-3, TIM-3) in single and combinatory treatment approaches has to be considered. Additional causes of immune escape will have to be identified in the future to recruit the full power of T-cell responses against B-lymphoblastic malignancies.

## MATERIALS AND METHODS

### Patients and bone marrow donors

After obtaining informed consent, PBMC and/or blasts of pediatric patients with ALL were used for functional analysis. Expression of co-signaling molecules on CD19^+^CD10^+^ bone marrow cells and expression of exhaustion markers on bone marrow T cells were analyzed on physiological bone marrow samples as compared to samples from pediatric ALL patients. T-cell function was analyzed by detecting proliferation (CFSE), intracellular Th1-cytokines, Perforin, Granzyme and CD107a expression.

A 12-year-old patient with B-precursor ALL who had relapsed after 2^nd^ allogeneic HSCT and did not respond to first cycle of Blinatumomab was treated with a combined treatment approach of Blinatumomab and Pembrolizumab (Keytruda®, Merck&Co, USA) in addition to CD45RA-depleted T cells from her haploidentical stem cell donor. Informed consent was given from the patients and the parents. Pembrolizumab was given as an off-label use. Inflammatory response during treatment was monitored by analysis of C-reactive protein (CRP), procalcitonin, ferritin, D-dimer, IL-6 and soluble IL-2 receptor (IL-2R) in peripheral blood of the patient. Flow cytometry was performed regularly to monitor blast load and T-cell count (CD3^+^CD4^+^ and CD3^+^CD8^+^) in peripheral blood. For evaluation of MRD, peripheral blood was analyzed and bone marrow puncture was performed on day 34 after treatment start.

### Functional analysis of T-cell proliferation and cytokine secretion to ex vivo stimulation with Blinatumomab

*Ex vivo* T-cell proliferation under addition of Blinatumomab was analyzed for healthy donors and patients (*in vivo* responders and non-responders to treatment with Blinatumomab) with carboxyfluorescein diacetate succinimidyl ester (CFSE) as described previously [33]. In brief, cells were labeled with 1.6μM CFSE (Molecular Probes; Invitrogen) and seeded (5×10^5^ cells/mL/ well) in 48-well plates with non-irradiated or irradiated (60 Gy) malignant cells (primary ALL-blasts, Raji, NALM-6, NALM-16, MHH-CALL-4). PBMC were exposed *ex vivo* to variable doses of Blinatumomab (Amgen Inc., Thousand Oaks, CA, USA) in a 37°C humidified incubator. Incubation time and effector/target cell ratios (E/T) were changed according to experimental requirements. After incubation period, cells were collected, followed by extracellular antibody staining and analyzed by flow cytometry. Controls were performed with 5×10^5^ CFSE-stained PBMC in co-culture with un-irradiated or irradiated malignant cells without addition of Blinatumomab. For functional analysis of cytokine secretion, PBMC were seeded with unirradiated or irradiated (60 Gy) target cells (Raji, NALM-6, NALM-16, MHH-CALL-4, patients' ALL blasts) in 48-well plates up to 48h and stimulated with Blinatumomab as described. Cells were counterstained by fluorochrome-labeled anti-CD3, anti-CD4 and anti-CD8 antibodies and flow cytometric assessment of IFN-γ^+^ and/or IL-2^+^ secretion of viable T cells was carried out by intracellular cytokine staining after addition of Brefeldin A (Sigma) for 4h. Subsequently, leukocytes were fixed with fix-and-perm solutions (Caltag Laboratories, Hamburg, Germany) according to the manufacturer's instructions.

### Impact of blocking antibodies to CD80 and CD86, PD-1 and/or CTLA-4 on T-cell proliferation and effector function

In order to evaluate the influence of co-signaling pathways for effector-target cell interactions, functional analysis of proliferation, IFN-γ and IL-2 secretion was performed after addition of blocking anti-human CD80 and anti-human CD86 antibodies (both Biolegend, San Diego, CA, USA) or anti-human PD-1 blocking antibody (Biolegend, San Diego, CA, USA) and/or CTLA-4 blocking human antibody Ipilimumab (Bristol-Myers Squibb GmbH & Co, New York, NY, USA). PBMC of healthy donors or ALL patients were seeded (5×10^5^ cells/well) in 48-well plates with irradiated (60 Gy) CD80^+^CD86^+^ Raji cells, patients' bone marrow ALL blasts or with CD80^−^CD86^−^ NALM-6 cells at an effector/target cell (E/T) ratio of 2:1, 10:1 or 100:1 for 48h. PBMC were stimulated with 100pg/ml or 1ng/ml Blinatumomab and incubated with 20μg/ml anti-CD80 and 20μg/ml anti-CD86 antibodies or 5μg/ml anti-PD-1 antibody and/or 10μg/ml Ipilimumab. Controls were performed with E/T cell co-cultures without addition of blocking antibodies and with unstimulated E/T cell co-cultures. After 48h, cells were collected without further restimulation, followed by intracellular and extracellular antibody staining as described above and analyzed by flow cytometry. Additional methods and details on functional assays are described in the Supplement.

Cytolytic activity was determined by a luciferase-based assay with NALM-6 expressing firefly luciferase-GFP as previously described as well as CD80 or PD-L1 expressing NALM-6 luciferase-GFP [45, 46] (kindly provided by Dr. Michel Sadelain, MSKCC, New York). PBMC and target cells were co-cultured at indicated E:T ratio and 1×10^5^ target cells/ well in black-walled 96-well plates in a duplicate or triplicate manner. 100ul/well luciferase substrate (Gold Biotechnology, USA) was added after 46 hours and light emission was determined by luminescence plate reader. Target cells were seeded alone in order to determine the maximal luciferase expression (relative light unit; RLU_max_) and lysis was determined as [1 − (RLUsample)/(RLUmax)] × 100. There was no relevant lysis detectable when PBMC and target cells were co-cultured without addition of Blinatumomab as compared to target cells alone. We demonstrated absence of lysis without Blinatumomab-stimulation in Figure 6 and compared Blinatumomab-induced lysis to same conditions (PBMC and target cells at same E:T ratio) without Blinatumomab for subsequent results.

### Expression of co-stimulatory and co-inhibitory molecules on ALL blasts, CD19^+^CD10^+^ bone marrow cells of controls and malignant CD19^+^CD10^+^ B-cell lines

Expression of co-signaling molecules PD-L1, PD-L2, PD-1, LAG-3, HVEM, BTLA, CD160, CD80, CD86, CD40, CD27, CD70, CD200, B7H3, B7H4, Galectin9, TIM-3, CD137L, CD278 (all: extracellular surface expression) and CTLA-4 (intracellular and extracellular expression) was analyzed by antibody staining and flow cytometry on ALL blasts and on physiologic CD19^+^CD10^+^ bone marrow cells of controls. In addition, expression of PD-L1, PD-1, LAG-3, HVEM, CD80, CD86, CD40, CD27, CD70 and CD200 was analyzed on CD19^+^CD10^+^ cell lines (Raji, NALM-6, NALM-16, MHH-CALL-4). Besides, PD-L1 expression on cell lines and patients' ALL bone marrow blasts was analyzed using intracellular flow cytometry as described above. Prior to analysis, functionality of all antibodies was tested on selected positive controls as previously described (s. Supplement) [12, 19, 34-44].

## SUPPLEMENTARY MATERIALS METERIALS METHODS FIGURES


