# Supplementary material for: T-cell responses against CD19+ pediatric acute lymphoblastic leukemia mediated by bispecific T-cell engager (BiTE) are regulated contrarily by PD-L1 and CD80/CD86 on leukemic blasts
Source: Oncotarget. 2016 Sep 30;7(47):76902–19. doi: 10.18632/oncotarget.12357 (PMC5363558; doi:10.18632/oncotarget.12357)
Supplement: Supplementary file 1 [file oncotarget-07-76902-s001.pdf]

T-cell responses against CD19$^+$ pediatric acute lymphoblastic leukemia mediated by bispecific T-cell engager (BiTE) are regulated contrarily by PD-L1 and CD80/CD86 on leukemic blasts

SUPPLEMENTARY METHODS

Culture media and additives

RPMI 1640 low endotoxin (Biochrom), supplemented with 1% L-glutamine (Biochrom) and 10% human pooled AB-serum (DRK Tübingen, Germany and DRK Ulm, Germany) was used for all cell cultures, except for maintaining cell lines. For cell lines Raji (human Burkitt's lymphoma cell line), MHH-CALL-4 and NALM-6 (both: human pre-B ALL cell lines) medium was supplemented with 10% fetal calf serum, 1% L-glutamine and 1% penicillin/streptomycin (all Biochrom). NALM-16 (human pre-B ALL cell line), THP-1 (human acute monocytic cell line) and SKBR3 cells (breast adenocarcinoma cell line, kindly provided by U. Hilcher, University Gynecological Hospital Tübingen) were cultured in RPMI 1640 with addition of 10% fetal calf serum and 1% penicillin/streptomycin (all Biochrom). NALM-6 expressing firefly luciferase-GFP were described previously (Brentjens et al., 2007). This line was subsequently retrovirally transduced to express CD80 (NALM-6-CD80) (Brentjens et al, 2003) or PD-L1 (NALM-6-PD-L1). Genetically modified cell lines were kindly provided by Dr. Michel Sadelain, MSKCC, New York.

Cell line authentication was performed by short tandem repeat (STR) analysis (DSMZ, Braunschweig, Germany) and confirmed full-matching STR reference profile with the respective parenteral/ reference line. IL-4, TNF-α, GM-CSF, IL-6 and IL-1β were provided by Cellgenix in research quality. For PD-L1 induction assays, IFN-γ (Imukin®) was provided by Boehringer and TNF-α by R&D Systems. Prostaglandin E was purchased from Sigma Aldrich.

Patients and bone marrow donors

All reported patients were treated at our institution between 2008 and 2015, and were registered with the local authorities of the University Children's Hospital, Tübingen, Germany. The treatment was conducted according to German ethical codes and regulations with the involvement of the Independent Ethics Committee of the University Children's Hospital, Tübingen, Germany, and in accordance with the provisions of the Declaration of Helsinki. All patients and their representative in law gave informed consent before Blinatumomab was started.

A 12-year-old patient with refractory B-ALL received combined treatment with Blinatumomab and Pembrolizumab (Keytruda®, Merck&Co, USA) in addition to CD45RA-depleted T cells from her haploidentical stem cell donor. CD45RA-depleted T cells were applied at a dose of 16.42 x 10^3 CD3/kg body weight. Pembrolizumab was administered the following day at a dose of 1mg/kg. The 2nd cycle of Blinatumomab was started one day later at a dose of 15µg/m^2/d without any previous steroid application. A second and third administration of Pembrolizumab (at doses of 1.25mg/kg and 2mg/kg) were given after 18 and 29 days, with additional CD45RA-depleted T cells from the same donor (12.23 x 10^3 CD3/kg) at day 29. Due to Philadelphia-chromosome like ALL with presence of INPP59-ABL1-fusion with tyrosine kinase sensitivity, the patient received the tyrosine kinase inhibitor Dasatinib. MRD was determined on day 34 after treatment start and Blinatumomab was ended two days later.

Expression of co-signaling molecules on CD19$^+$CD10$^+$ bone marrow cells and expression of exhaustion markers on bone marrow T cells were analyzed on physiological bone marrow samples as compared to patients' samples. All patients and controls gave informed consent to use remnant material of bone marrow aspirates for further analyses. Material for analyses was only obtained in case of indicated bone marrow harvest. Bone marrow harvest from controls was performed due to therapeutic issues of patients.

Functional analysis of Blinatumomab-induced T-cell responses

For functional T-cell assays, stimulation of PBMC with Staphylococcus enterotoxin B (SEB) (Sigma-Aldrich) was used as positive control. All flow cytometric analyses were performed on a LSRII cytometer using FACS-DIVA Vers. 6.1.3 (all BD Bioscience) and Flow Jo software (TreeStar, Ashland, OR, USA).

Cytotoxicity was analyzed by flow cytometric CD107a expression after 4-6h stimulation of 1x10^6 PBMC with 1x10^5 irradiated Raji cells (60 Gy) and Blinatumomab in the presence of a CD107a antibody and MonensinA (BD Bioscience). Granzyme-B (GrB) and Perforin staining was performed intracellularly after 24-48h stimulation of 1x10^6 PBMC with 1x10^5 irradiated Raji cells and Blinatumomab. As described, for intracellular analysis, Brefeldin A (Sigma) was added for 4h and leukocytes were fixed with fix-and-perm solutions (Caltag Laboratories, Hamburg, Germany) according to the manufacturer's instructions.

Antibodies

Flow cytometry of cells was performed using saturating conditions of the following antibodies: anti-CD3 PE-Cy7, anti-CD3 PE-CF594, anti-CD4-APC-H7, anti-CD4-PE, anti-CD8 FITC, anti-CD8 PE, anti-IFN-γ PE, anti-IL-2 APC, anti-CD19 APC, anti-CD19 APC-H7, anti-CD19 FITC, anti-CD19 PE, anti-CD40 FITC, anti-B7H4 PE, anti-CD80 PE, anti-CD80 PE-Cy7, anti-CD80 V450, anti-CD83 FITC, anti-CD27 APC, anti-ICOS (CD278) BV421, anti-CTLA-4 APC, anti-CD107a APC, anti-GranzymeB FITC (all BD Bioscience), anti-CD10 PE-Cy7, anti-CD8 BV570, anti-CD8 BV605, anti-CD16 AF700, anti-CD20 PerCp, anti-CD86 BV421, anti-CD56 BV421, anti-CD70 PE, anti-PD-L1 APC, anti-CD200 BV421, anti-PDL2 PE, anti-Galectin-9 APC, anti-TIM-3 BV421, anti-CD137L PE, anti-HVEM PE, anti-B7H3 PE, anti-BTLA APC, anti-CD160 PE, anti-PD-1 BV421, anti-PD-1 APC, anti-CD107 PE-Cy7, anti-Perforin AF647, anti-IFN-γ BV785 (all Biolegend), anti-LAG3 PE (R&D Systems) and anti-CD8 PB (Dako). Dead cells were excluded by staining with the amin-reactive dye Alexa Fluor 350 (AF350; Invitrogen), Pacific Blue Succinimidyl Ester (Life Technologies) or viability dye eFluor780 (eBioscience).

Functionality of antibodies was tested as follows: Expression of CD40, CD86, CD80 and CD70 was tested on Raji cells, expression of B7H4 on SKBR3 cells, expression of CD83, PD-L1, CD200, PD-L2, Galectin-9, TIM-3, HVEM, CD137L, B7H3 and CD80 on generated DCs, expression of BTLA on $CD19^+$ cells, expression of CD160 on $CD8^+$ T cells and expression of CD27 on $CD3^+$ T cells. Expression of PD-1, ICOS (=CD278), LAG-3 and CTLA-4 was determined on SEB-stimulated T cells after 48h. Expression of co-signaling molecules was tested as compared to isotype and FMO controls.

Expression of T-cell exhaustion markers

Expression of T-cell exhaustion markers PD-1, TIM-3 and LAG-3 on bone marrow T cells of patients or controls was analyzed by extracellular antibody staining and flow cytometry. Analysis was performed directly *ex vivo* and after 48h-stimulation of bone marrow cells (at least $5x10^5$ cells/well) with 1ng/ml Blinatumomab. Stimulation with 10µg/ml SEB was performed as positive control. Dose-dependent expression of CTLA-4 and PD-1 on T cells was further analyzed after 48h-72h incubation of PBMC with irradiated Raji cells and stimulation with various concentrations of Blinatumomab. Target cell-dependent expression of PD-1 was additionally analyzed on T cells of healthy donors after 48h-incubation of PBMC with different target cell lines (MHH-CALL-4, Raji, NALM-6, NALM-16) at an effector/target cell ratio of 2:1 and stimulation with 1ng/ml Blinatumomab. CTLA-4 expression was detected by intracellular antibody staining as described above and PD-1 expression was determined by extracellular antibody staining and flow cytometry.

Induction of PD-L1 expression by TH1 cytokines IFN-γ and TNF-α

Induction of PD-L1 expression on cell lines (Raji, NALM-6, NALM-16 and MHH-CALL-4) and on patients' ALL blasts was analyzed after 24-48h incubation of cells (at least $1x10^5$ cells/well in 48-well plates) with 100ng/ml IFN-γ (Imukin®, Boehringer Ingelheim, Ingelheim, Germany) and 100ng/ml IFN-γ+10ng/ml TNF-α (R&D Systems, Minneapolis, MN, USA). Unstimulated samples, isotype control, FMO control and induced PD-L1 expression on THP-1 cells after stimulation with cytokines IFN-γ and IFN-γ+TNF-α were used as controls. Surface expression was performed by extracellular antibody staining and flow cytometry. Mean fluorescence intensity (MFI) of PD-L1 staining was determined on gated live $CD19^+CD10^+$ cells (or on gated live cells in case of THP-1 cells) and compared to background (isotype/ FMO) MFI. Induction of PD-L1 was defined as positive when MFI after stimulation with cytokines IFN-γ or IFN-γ and TNF-α was ≥10% above the MFI in unstimulated samples.

Analysis of PD-L1 expression by immunohistochemistry

Immunocytochemical staining of PD-L1 was applied for native material (1:250 native material, rabbit monoclonal E1L3N; Cell Signaling, Danvers, MA 01923, USA). Unfixed cytologic smears were air-dried overnight and subsequently fixed with paraformaldehyde for 30min. Multimere Optiview (Roche, Mannheim, Germany) detection was performed without pre-treatment, the antibody was incubated at 37° for 32 min. The immunostaining was performed on the automated immunostainer Ventana Benchmark (Roche/Ventana Medical Systems Inc., Tucson, AZ, USA) according to the manufacturer's instructions.

Statistical analysis

Statistical analysis was done with the Mann–Whitney test and paired t test using Graph Pad Prism software 6.05 (GraphPad Software, La Jolla, CA). All p values are two-tailed and statistical significance was defined as $p < 0.05$.

SUPPLEMENTARY FIGURES

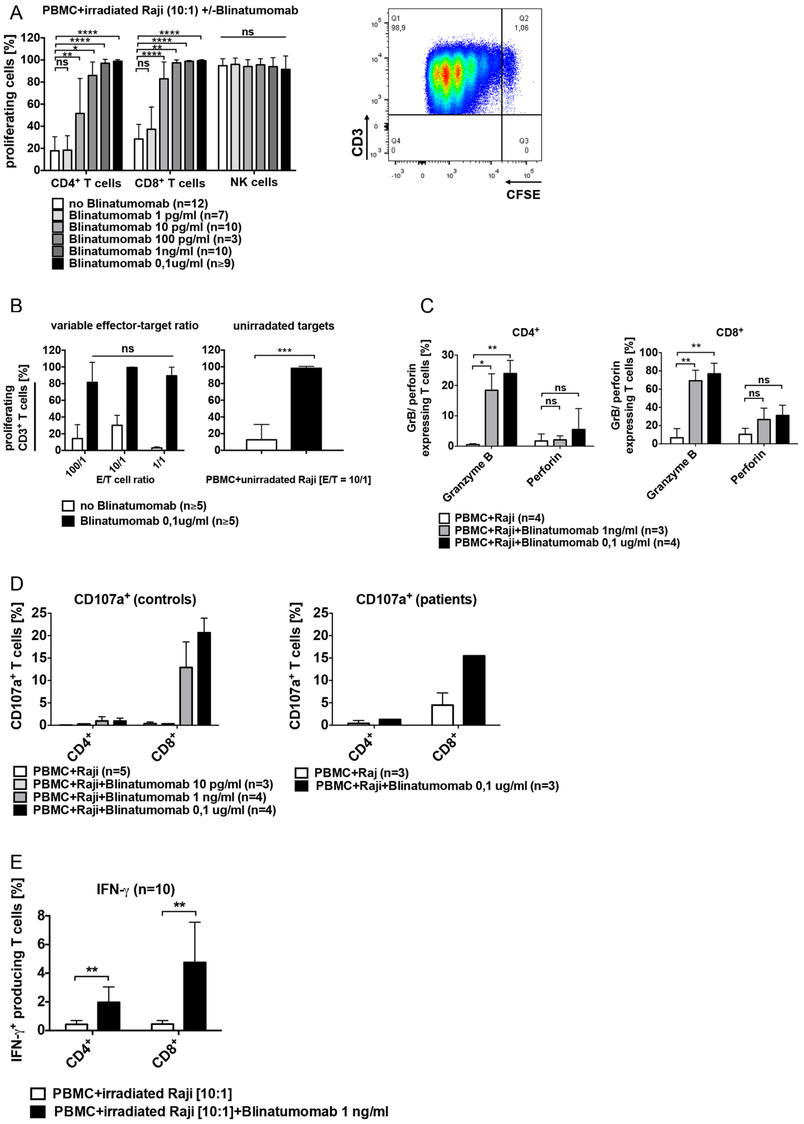

Supplementary Figure S1: Proliferation capacity and effector function of CD4$^+$ and CD8$^+$ T cells under stimulation with Blinatumomab. A. PBMC of healthy donors were incubated with irradiated Raji cells (effector/target cell ratio=10/1) and different doses of Blinatumomab. Proliferation of CD4$^+$ and CD8$^+$ T cells as compared to NK cells (CD56$^+$CD16$^+$) was determined by CFSE assay after 5 days. Bars show mean and SD. *p < 0.05, **p < 0.01, ****p < 0.0001, paired t test. The right plot demonstrates a flow cytometric example of CD3$^+$ T-cell proliferation after stimulation of PBMC with irradiated Raji cells and 0.1 µg/ml Blinatumomab. **B.** PBMC of healthy donors were incubated with Raji cells for 5 days. Proliferation capacity of CD3$^+$ T cells after incubation with irradiated Raji cells at different effector/target cell ratios with and without addition of Blinatumomab (left plot) (n=6). Blinatumomab-induced proliferation capacity of CD4$^+$ and CD8$^+$ T cells when unirradiated Rajis were used as targets at an effector/target cell ratio of 10:1 (n=5; ***p < 0.001, paired t test). **C.** Granzyme B and Perforin expression of CD4$^+$ and CD8$^+$ T cells after 24-48h incubation of PBMC of healthy donors with irradiated Raji cells (effector/target cell ratio=10/1) and different doses of Blinatumomab (*p < 0.05, **p < 0.01, paired t test). **D.** PBMC of healthy donors or patients receiving Blinatumomab treatment were incubated with irradiated Raji cells and stimulated with different doses of Blinatumomab. Dose-dependent CD107a expression of CD4$^+$ and CD8$^+$ T cells was determined after an incubation period of 4-6 hours. **E.** Expression of pro-inflammatory Th1 cytokine IFN-γ after stimulation of PBMC (healthy donors) with irradiated Raji cells and Blinatumomab as compared to control cytokine secretion without addition of Blinatumomab. Cytokine production of CD4$^+$ and CD8$^+$ T cells was determined by flow cytometry after an incubation period of 14-17 hours (**p < 0.01, paired t test).

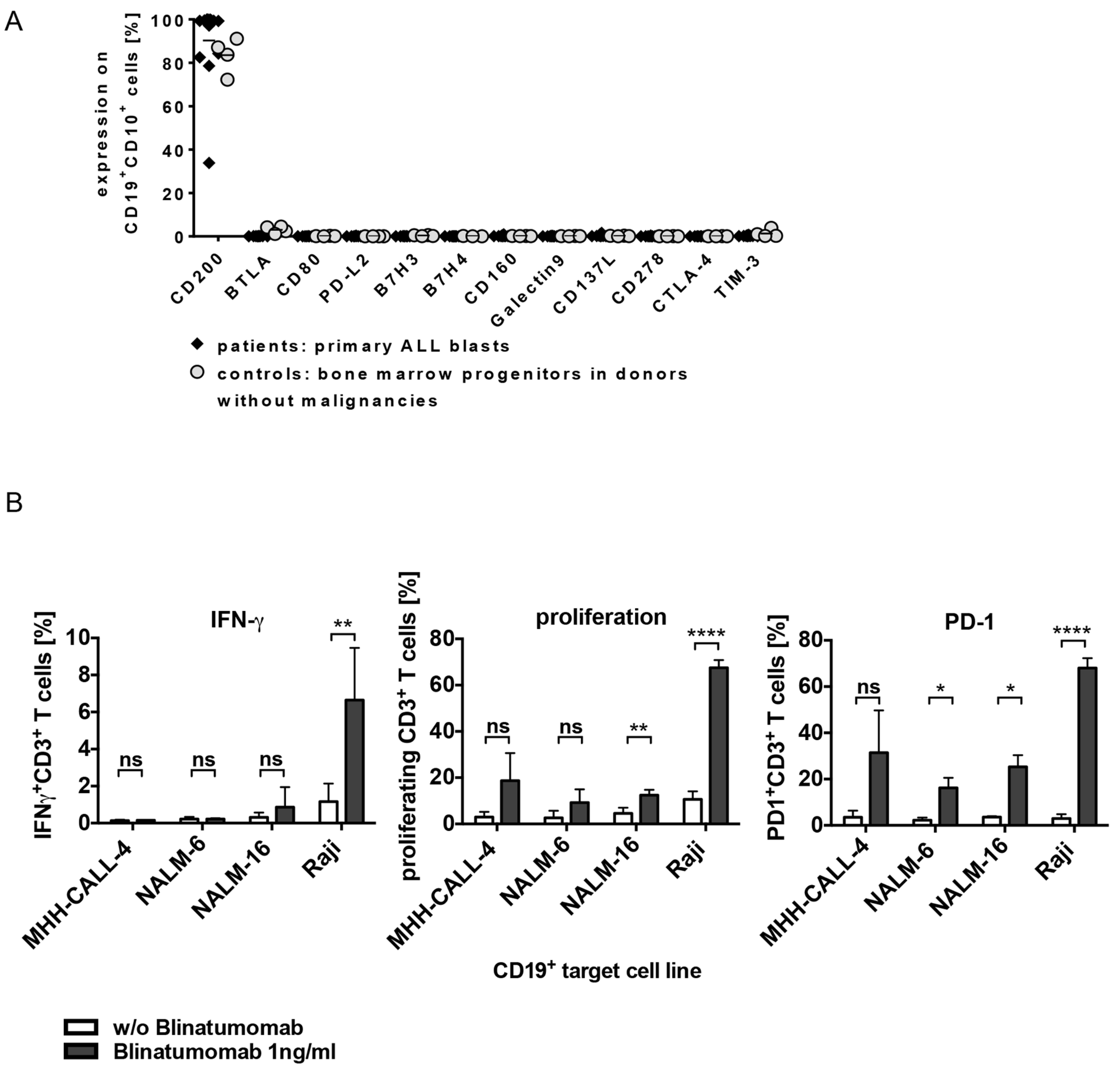

Supplementary Figure S2: A. Surface expression of co-signaling molecules on CD19$^+$CD10$^+$ ALL blasts. Patients' blasts were screened by flow cytometry for surface expression of further co-stimulatory and co-inhibitory molecules as compared to expression on physiologic CD19$^+$CD10$^+$ bone marrow cells as controls. CD200: n=13 (patients) and n=4 (controls), BTLA: n=10 (patients) and n=4 (controls), CD80: n=10 (patients) and n=4 (controls), PD-L2: n=15 (patients) and n=4 (controls), B7H3: n=10 (patients) and n=4 (controls), B7H4: n=10 (patients) and n=3 (controls), CD160: n=10 (patients) and n=4 (controls), Galectin9: n=10 (patients) and n=4 (controls), CD137L: n=10 (patients) and n=4 (controls), CD278: n=10 (patients) and n=4 (controls), CTLA-4: n=10 (patients) and n=4 (controls), TIM3: n=10 (patients) and n=4 (controls). **B.** Target cell-dependent IFN-γ secretion, proliferation and PD-1 expression of CD3$^+$ T cells after stimulation with Blinatumomab. PBMC of healthy donors were incubated with irradiated Raji, NALM-6, NALM-16 or MHH-CALL-4 cells (effector/target cell ratio: 10/1). IFN-γ secretion (MHH-CALL-4: n=4, NALM-6: n=3, NALM-16: n=4, RAJI: n=5), PD-1 expression (MHH-CALL-4: n=3, NALM-6: n=3, NALM-16: n=3, RAJI: n=5) and proliferation (MHH-CALL-4: n=3, NALM-6: n=3, NALM-16: n=3, RAJI: n=5) were determined after 48h-stimulation with Blinatumomab 1ng/ml. Unstimulated samples served as controls. Bars represent data from independent experiments and mean and SD are indicated. $*p < 0.05$, $**p < 0.01$, $****p < 0.0001$, paired t test.

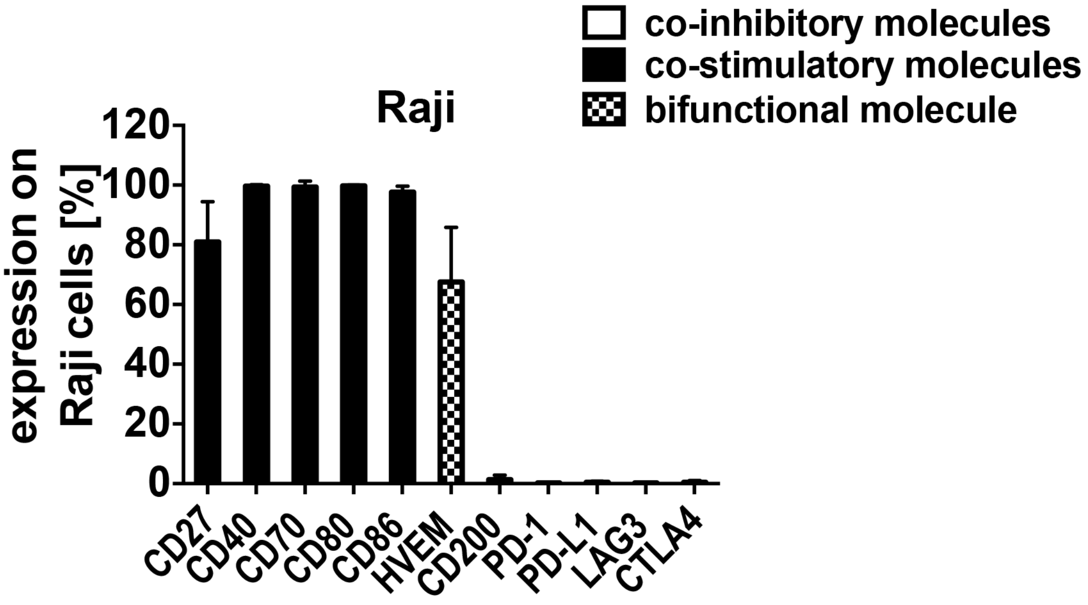

Supplementary Figure S3: Surface expression of co-stimulatory and co-inhibitory markers on CD19$^+$CD10$^+$ Raji cells. Raji cells (≥n=4, max. n=11) were screened for surface expression of relevant co-inhibitory and co-stimulatory molecules by flow cytometry.
